# 18F-5-fluoro-aminosuberic acid PET/CT imaging of oxidative-stress features during the formation of DEN-induced rat hepatocellular carcinoma

**DOI:** 10.7150/thno.100467

**Published:** 2025-01-01

**Authors:** Feng Xiong, Yilin Yang, Zhengru Han, Buchuan Zhang, Kijung Kwak, Pei Wang, Qiaorong Chen, Ziqiang Wang, Jingfei Yang, Xiaoyun Deng, Sijuan Zou, Zhuoli Zhang, Pengtao You, Bo Yu, Xiaohua Zhu

**Affiliations:** 1 Department of Nuclear Medicine, Tongji Hospital, Tongji Medical College, Huazhong University of Science and Technology, Wuhan, China.; 2 Department of Nuclear Medicine, Tongji Hospital, Tongji Medical College and State Key Laboratory for Diagnosis and Treatment of Severe Zoonotic Infectious Diseases, Huazhong University of Science and Technology, Wuhan, China.; 3 Hubei University of Chinese Medicine, Wuhan 430030, Hubei Province, China.; 4 University of Maryland School of Medicine, 655 W Baltimore St S, Baltimore, MD 21201, USA.; 5 Department of Radiological Sciences, University of California Irvine, CA, USA.

**Keywords:** [18F]FASu, PET imaging, Oxidative stress, SLC7A11, HCC

## Abstract

**Rationale:** The role of oxidative stress metabolism during hepatocellular carcinoma (HCC) formation potentially allows for positron emission tomography (PET) imaging of oxidative stress activity for early and precise HCC detection. However, there is currently limited data available on oxidative-stress-related PET imaging for longitudinal monitoring of the pathophysiological changes during HCC formation. This work aimed to explore PET-based longitudinal monitoring of oxidative stress metabolism and determine the sensitivity of [18F]-5-fluoroaminosuberic acid ([18F]FASu) for assessing pathophysiological processes in diethylnitrosamine (DEN) induced rat HCC.

**Methods:** Genomic and clinical data were obtained from the HCC dataset (n = 383) in The Cancer Genome Atlas (TCGA-LIHC) and Gene Expression Omnibus (GEO) datasets. Wistar rats were administered DEN weekly, either by gavage (i.g.) at doses of 10 mg/kg or 80 mg/kg or by intraperitoneal injection (i.p.) at 80 mg/kg, with continuous modeling over a 12-week period followed by 24 weeks of consecutive feeding. PET/CT imaging was conducted at weeks 8, 15, and 21 by tail vein injections of [18F]FASu and [18F]FDG (~3.7 MBq). Finally, contrast-enhanced CT imaging of the nodules was performed at the designed time point. The rats in each group were sacrificed at multiple time points to perform a correlation analysis between PET imaging findings and histological examinations.

**Results:** Bioinformatics analysis revealed that upregulation expression of SLC7A11 in HCC indicates oxidative stress-altered cellular metabolism and allows early detection of HCC formation. By simulating different levels of oxidative stress in DEN-induced rat HCC, the SUVmax of [18F]FASu PET imaging positively correlated with the expression of CD44 and SLC7A11 (r = 0.7913, *P* < 0.0001; r = 0.7173, *P* < 0.0001, respectively), which maintain redox homeostasis in the cells. Compared with 18F-fluorodeoxyglucose ([18F]FDG), [18F]FASu PET imaging demonstrated higher sensitivity for HCC diagnosis and enabled the characterization of pathological changes in DEN-induced rat HCC at an early stage.

**Conclusions:** Our findings regarding the oxidative stress characterization of HCC formation in DEN-induced rat models using [18F]FASu PET imaging demonstrated the exciting potential of oxidative-stress-related PET imaging for monitoring the pathophysiological changes during HCC formation.

## Introduction

Hepatocellular carcinoma (HCC) currently stands as the second leading cause of cancer-related deaths worldwide 1. Regrettably, the average 5-year survival rate for HCC remains below 20% due to a high prevalence of diagnoses at advanced stages 2. Early and precise detecting of the tumor and its progression is therefore crucial. Recently, various features of cancer cells, including metabolic processes or biomarkers, have been identified in malignant neoplasms using noninvasive positron emission tomography (PET) imaging. For instance, 18F-fluorodeoxyglucose ([18F]FDG), a typical PET tracer identifying abnormal glucose metabolism in cancers, has been valuable in detecting certain malignancies 3. However, in the case of HCC, [18F]FDG has limited use due to inadequate sensitivity 4, 5. The uptake of [18F]FDG in malignant tumors largely depends on the presence of facilitated glucose transporters, including type 1 (Glut 1) which is rarely expressed in HCC 6, 7. Hence, the sensitivity of [18F]FDG PET for imaging HCC is low, and previous studies reported a range from 40% to 68% 8, 9. For well-differentiated HCC, [18F]FDG testing is less positive than for poorly differentiated 10. In addition to the increased glucose metabolism, increased lipid-related demand in HCC allows lipophilic PET imaging tracers [11C]-acetate, [18F]-fluorocholine, and [11C]-choline to accumulate in the liver and result in low-contrast images. Another class of radiotracers, immunoPET tracers, target biomarkers' expression and can further improve the specificity. Especially, antibody or nanobody-based GPC3-targeted tracers and the PDGFRβ-targeted trimeric affibodies have strong avidity for well- and moderately differentiated HCCs but are limited by their complexity in preparation and high off-target accumulation in liver and kidney 11. Recent studies of fibroblast activating protein (FAP) targeted ligands showed superior diagnostic accuracies in detecting HCC, but false positives due to postoperative stromal fibrosis were noted 12. Radiolabeled prostate-specific membrane antigen (PSMA)-targeted derivatives 13, 14, previously thought to be specific to prostate cancer, have also demonstrated avidity to HCC. Lastly, while early and more advanced HCC have been reported to be characterized by neovascularization, the mechanism of this process is still unclear, and further robust evidence is needed to validate its diagnostic value. Thus, the question of how to diagnose HCC with noninvasive PET imaging has yet to be answered and remains an unmet need in clinics. This predicament suggests exploring other optional targets of PET imaging and deepening the correlation analysis between selected targets and PET imaging features during the formation of HCC.

HCC usually develops from chronic liver injuries associated with oxidative stress over a long period 15. The above etiology and the chronic nature of HCC formation indicate the potential of establishing specific features of oxidative-stress metabolism during HCC formation for early diagnosis, emphasizing the importance of animal models for longitudinal characterization during HCC formation. Solute carrier family 7a member 11 (denoted as SLC7A11; also called xCT) mediates the cellular uptake of cystine in exchange for intracellular glutamate at the plasma membrane and plays a critical role in protection against oxidative stress and maintains redox homeostasis in the cells 16. It was found that substances like Erastin, which pharmacologically impede SLC7A11-mediated cystine absorption, cause a novel type of cell death known as ferroptosis 17. Glutathione peroxidase 4 (GPX4), a member of the GPX family, is a major defense mechanism that cells have evolved to detoxify adverse lipid peroxides produced during ferroptosis. This process is pivotal in safeguarding cells against oxidative stress and preserving redox homeostasis within them 18. In tumors, the increased expression and activity of SLC7A11 potentially offer a survival advantage by enabling access to cysteine, the rate-limiting precursor for glutathione (GSH) synthesis, via the more abundant extracellular cystine. Thus, SLC7A11 targeting PET imaging exhibited the potential to delineate various tumors, offering an exciting target marker for HCC diagnosis 19. However, the heterogeneity of SLC7A11-targeting tracer retention in tumors further requires more understanding of the correlation between SLC7A11 expression and PET imaging features during the formation of HCC.

Animal models provide a useful route for determining the biology and image features of HCC. Baek *et al.* reported PET imaging of SLC7A11 as a promising tool for orthotopic HCC detection in rats 20. However, animal models of HCC, particularly those inducing fibrosis and cirrhosis, are essential for detecting tumor initiation and progression close to clinical cases 21, 22. One such model is diethylnitrosamine (DEN)-induced rat HCC, which has been shown to mimic human liver fibrosis, cirrhosis, and subsequent complete hepatocellular carcinogen 23, 24. DEN is an indirect alkylating agent that induces oxidative stress by producing reactive oxygen species, increasing the risk of tumor formation in various organs such as the liver, skin, gastrointestinal tract, and respiratory system 25. Although plenty of DEN-induced HCC was reported, to our knowledge, no research has introduced oxidative-stress-related PET imaging for the longitudinal characterization of the pathophysiological changes during tumor formation in the DEN-induced rat HCC. No doubt, this information will strengthen the potential oxidative-stress-related PET imaging for HCC diagnosis.

Herein, 18F-5-fluoro-aminosuberic acid (denoted as [18F]FASu), a new 18F-labeled ʟ-cysteine derivative that can be taken up by the SLC7A11, was used as SLC7A11-targeting PET tracer 26. In this work, we aimed to explore PET-based longitudinal monitoring of oxidative stress metabolism and determine the sensitivity of [18F]FASu for assessing pathophysiological processes in DEN-induced rat HCC.

## Methods

### [18F]FASu synthesis

[18F]FASu was synthesized as previously reported 27. Briefly, [18F]fluoride was trapped by a Sep-Pak light QMA cartridge and eluted by a mixture of Kryptofix[2.2.2] (10 mg in 200 mL acetonitrile) and K_2_CO_3_ (2 mg in 200 mL H_2_O). After drying, Kryptofix [2.2.2]/18F- was reacted with the precursor (1.5 mg in 500 ml of DMSO) for 20 min at 95°C. After the reaction was completed, it was cooled to room temperature and then diluted with 8 ml of H_2_O before loading onto a Waters tC18 Plus Sep-Pak. 18F- was washed down using a large amount of water and then was eluted with 2 ml of acetonitrile. After drying again, 0.5 mL of trifluoroacetic acid and 0.01 mL of anisole were added and reacted at 95°C for 7 min to dissociate the protecting groups. Similarly, 0.5 mL of H_2_O was used to pass the column after the third drying, and 0.5 mL of H_2_O was again used to rinse the residual product off the column. All drying steps are under the flow of N_2_ at 95℃.

The final product was determined by radio-HPLC and was stable *in vitro* for 4h without significant metabolite production (**Figure** S1). Only one chiral compound was used in this experiment (ʟ-[18F]FASu: ᴅ-[18F]FASu = 87:13, **Figure** S2A). Decay-corrected radiochemical yield (RCY) was 16% ± 7% (n = 4), and radiochemical purity was greater than 99%, and molar activity was 23 ± 5 GBq/mmol (n = 4).

### Animal models

All the animal experiments were performed in compliance with the approved protocol. To induce the primary HCC, Wistar rats (male, 150-200 g, Week 6) were fed with 10 mg/kg or 80 mg/kg of DEN by gavage (i.g.) and 80 mg/kg by intraperitoneal injection (i.p.), respectively, with continuous modeling for 12 weeks followed by 24-week consecutive feeding. Tissue samples from animals were prepared as 10-µm-thick slices for pathological analysis of hematoxylin & eosin (H&E) or alpha-fetoprotein (AFP) immunohistochemical staining. The experimental procedures and the rats' feeding schedule were approved by the Institutional Animal Care and Use Committee of Tongji Hospital, Tongji Medical College, Huazhong University of Science and Technology, China.

### Bioinformatics analysis

The mRNA levels of SLC7A11 in HCC patients and clinical data in the TCGA-LIHC database were acquired from the Xena (https://xenabrowser.net/heatmap/#). Analysis of SLC7A11 mRNA expression levels in HCC patients and DEN-induced rat models were selected by GSE144269, GSE141090, and GSE182860 from the GEO database (National Center for Biotechnology Information (nih.gov)). In addition, datasets GSE144269 comprising 70 HCC tissues and normal liver tissues derived from patients were also analyzed to detect the relative expression of SLC7A11 genes in HCC tissues. As for DEN-induced rat models, datasets GSE141090 contains 3 each of hepatitis (LH), liver cirrhosis (LC), control groups (NC), and hepatocellular carcinoma (HCC), and datasets GSE182860 contains 4 samples of HCC and 4 samples of normal liver tissue from DEN-induced rats at Week 8.

### Immunohistochemical assay

Paraffin-embedded, formaldehyde-fixed tumor sections underwent rehydration, antigen retrieval, and blocking with 5% bovine serum albumin (BSA) in TBST before being incubated with various antibodies: SLC7A11 (1:300; Novus Biologicals, NB300-318), AFP Polyclonal (1:300; Proteintech, 14550-1-AP), anti-CD44 (1:8000; Abcam, ab189524), anti-iNOS (1:400; Boster, BA0362), 8-oxo-dG antibody (1:750; R&D Systems, 4354-MC-050), γ-H2AX antibody (1:200; Cell Signaling Technology; 1:200) and Ki-67 (1:800; Cell Signaling Technology, 9129). Positive staining was visualized using the 3,3'-diaminobenzidine (DAB) reaction, followed by counterstaining with hematoxylin and imaging under a bright-field microscope. The details are supplied in the supplemental materials.

### PET imaging and computed tomography (CT) imaging

Rats were intravenously injected with 3.70 MBq of [18F]FASu or 1.85 MBq [18F]FDG (in-house PET center, Tongji Hospital, Tongji Medical College, Huazhong University of Science and Technology, Wuhan, China) respectively. A 10 min acquisition was performed 1 h later on a PET/CT scanner (uBio-EXPLORER, United Imaging, China). Data were reconstructed to get more high-resolution images using an ordered subsets expectation maximization (OSEM) algorithm (4 OSEM iterations, resolution: 1.4 mmHD) with scatter, attenuation, and decay corrections applied.

Non-contrast CT and contrast-enhanced CT (CECT) were performed on Rats at Week 22 using the CT module of u-EXPLORER PET/CT (United Imaging) with the following scan and reconstruction parameters: 120 kV, 80 mA, axial field of view 30 cm, matrix 512 × 512, and slice thickness 2mm with overlap scan. During imaging, the rats were anesthetized by 2% isoflurane inhalation. A non-contrast CT scan was performed before a "bolus-like" administration of 1.2 ml iodixanol (160 mg of iodine/ml) via one of the rat tail veins. Subsequently, five scans were performed at 10, 20, 35, 50, and 90 s after the administration.

All non-contrast CT and CECT images were analyzed using a self-contained u-EXPLORERPET/CT (United Imaging) workstation. The CT values of lesions were measured by manually delineating the region of interest.

### Statistics

The statistical analysis was performed using version 8.0 of GraphPad Prism. All *in vitro* data were collected on various days from three or more distinct biological replicates, and the mean value was provided with one standard deviation (SD). To determine statistical significance, a two-tailed, unpaired Student's t-test was employed. One-way analysis of variance (ANOVA) was used for comparisons involving multiple samples, and this was followed by t-tests and the multiple comparison correction technique (also known as Tukey's method). A log-rank (Mantel-Cox) test was used to evaluate the Kaplan-Meier survival curves. Crude P-values in multiple comparisons were adjusted using the Holm-Bonferroni method to control the family-wise error rate. In every analysis, a P-value of less than 0.05 was deemed statistically significant. Additionally, Receiver Operating Characteristic (ROC) analysis was conducted, including the evaluation of the Area Under the Curve (AUC). Using the Youden Index, diagnostic accuracy was evaluated at cut-off points established in the current investigation.

## Results

### Upregulation of SLC7A11 in HCC

**Figure 1:**
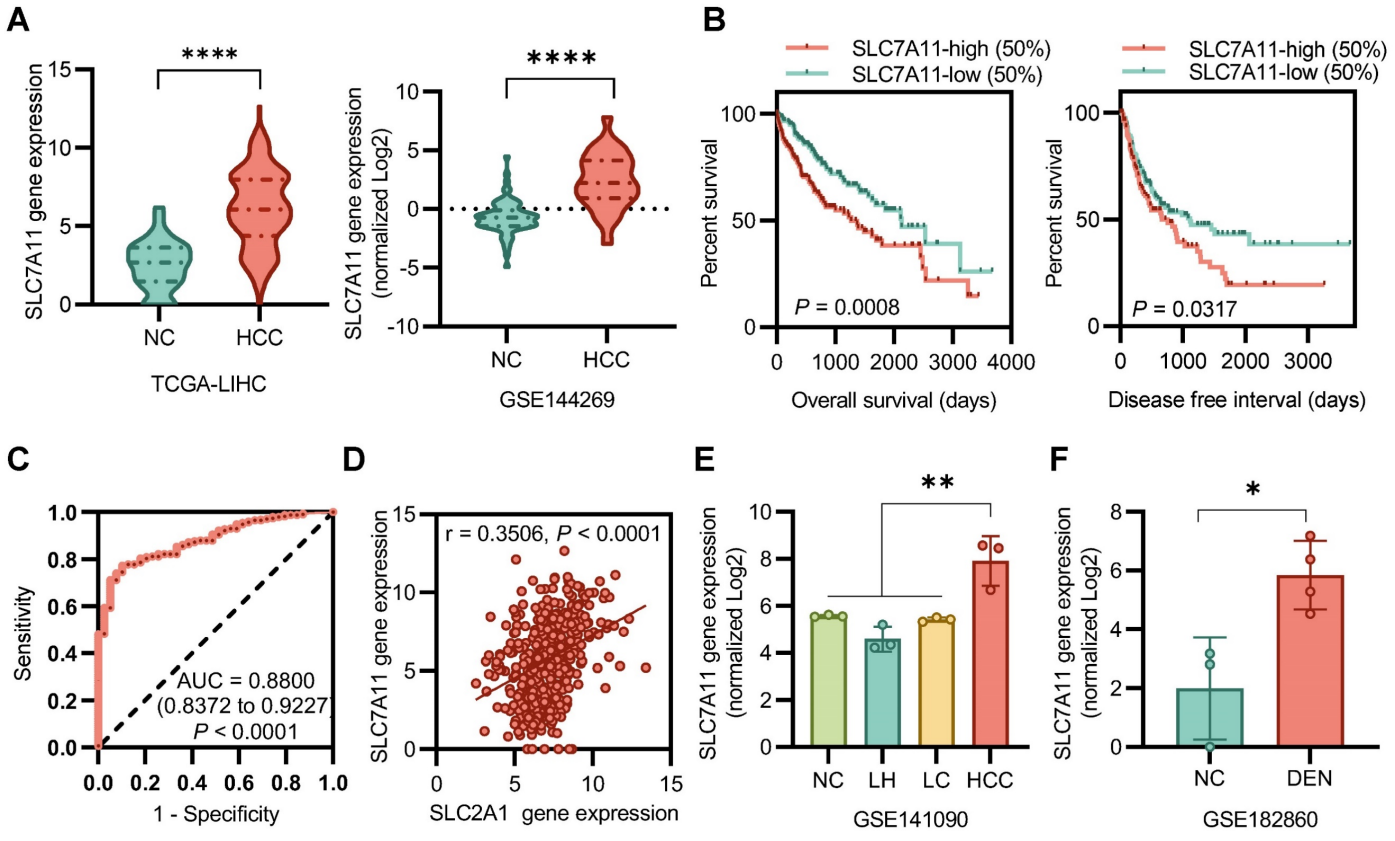
SLC7A11 is upregulated in HCC. **(A)** Gene expression levels of SLC7A11 were upregulated in tumor tissues derived from HCC patients. **(B)** Poor prognosis with high SLC7A11 of overall survival (OS) and disease-free interval (DFI). **(C)** An ROC curve showed that SLC7A11 could effectively distinguish HCC from para-cancer tissues. **(D)** There was no significant correlation between the expression of SLC7A11 and SLC2A1 in all patients. **(E)(F)** The mRNA expression of SLC7A11 in HCC is higher than that of other types of liver diseases in DEN-induced rat models. LH = liver hepatitis; LC = liver cirrhosis; NC = normal control; HCC = hepatocellular. *P < 0.05. *P < 0.01. ***P < 0.0001.

SLC7A11 mRNA levels were significantly higher in HCC tissues than in normal tissues (**Figure** 1A). Kaplan-Meier analysis determined that patients' overall survival (OS) and disease-free interval (DFI) were associated with the expression of SLC7A11 in tumors. Patients were stratified by SLC7A11 expression into high (upper 50)- or low (lower 50)- expression groups. Notably, patients with tumors expressing high levels of SLC7A11 exhibited a significantly shorter OS (*P* < 0.001) and DFI (*P* < 0.05) (**Figure** 1B). An ROC curve showed that SLC7A11 could effectively distinguish HCC from para-cancer tissues (**Figure** 1C). Correlation analysis between SLC7A11 and SLC3A2 in all patients in the TGCA-LIHC database (r = 0.3506, *P* < 0.0001)) suggests a mismatch between [18F]FASu and [18F]FDG uptake (**Figure** 1D). For the DEN-induced rat model database, we found that the expression level of the SLC7A11 gene was higher in HCC than in liver hepatitis (LH), liver cirrhosis (LC), and normal control (NC) (**Figure** 1E). In a time-dependent manner, the expression level of SLC7A11 was significantly higher than that of the control group in the DEN-induced 8 weeks (**Figure** 1F). Overall, SLC7A11 was closely associated with HCC, was significantly upregulated in HCC patients and DEN-induced rat models and was affected by the induction time.

### Effect of DEN administration methods on HCC formation

**Figure 2:**
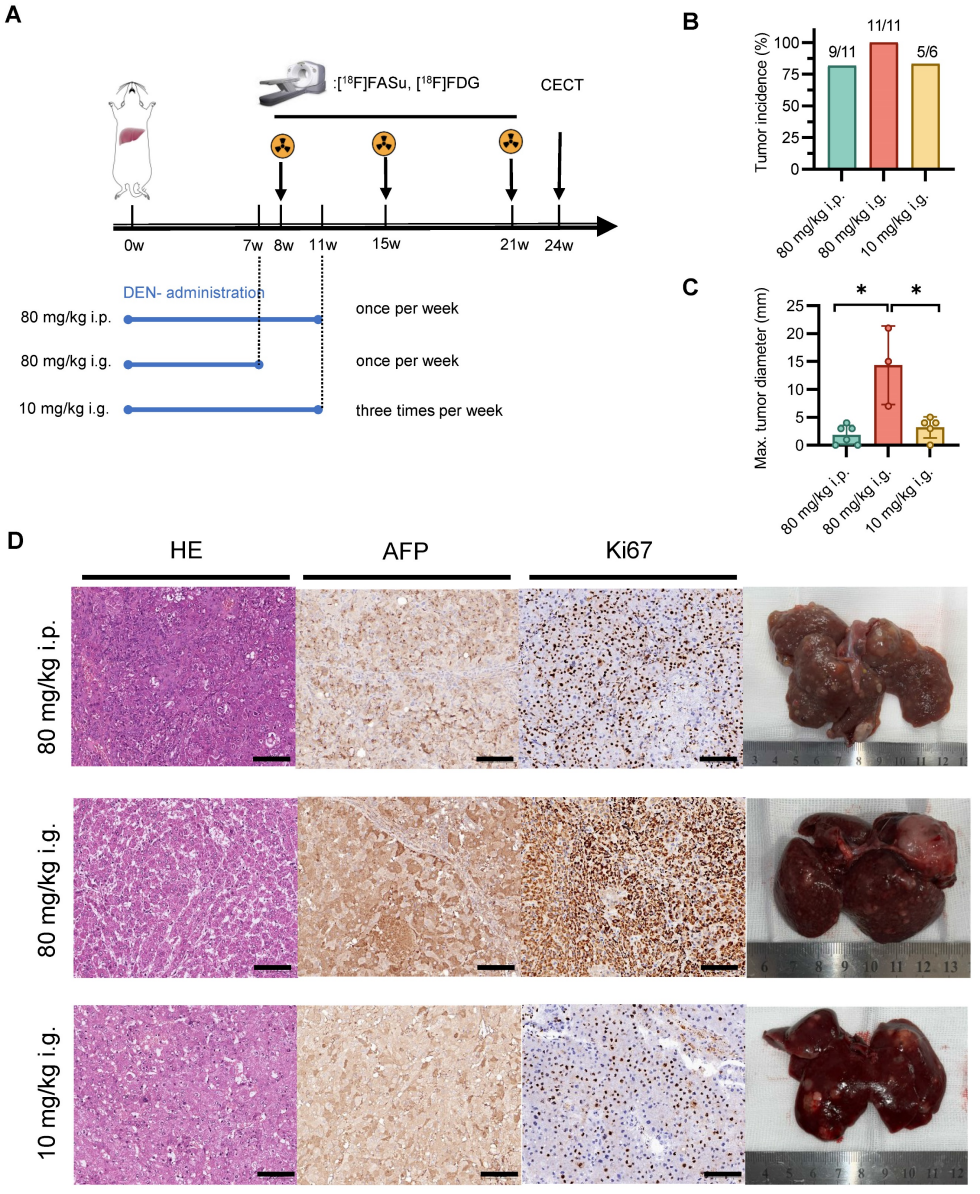
Effect of different modes of drug administration on HCC formation. **(A)** Timeline protocol to characterize DEN-induced rat model of HCC (three groups: group 80 mg/kg i.p., n = 11; group 80 mg/kg i.g., n = 11; and group 10 mg/kg i.g., n = 6). [18F]FASu, [18F]FDG PET/CT and contrast-enhanced CT (CECT) were performed at Week 8, Week 15, Week 21 and Week 24, respectively. **(B)** Comparison of tumor induction success rates at Week 15 after DEN injection. **(C)** Average maximal diameters of tumors compared in different groups (80 mg/kg i.p.: 2.75 ± 1.26, n = 4; group 80 mg/kg i.g.: 14.33 ± 7.02, n = 3; and group 10 mg/kg i.g.: 4.00 ± 0.82, n = 4). **(D)** Representative microscopic features of HCC in H&E stained and AFP and Ki67 immunohistochemical stained liver sections from rats. Photographs showing the characteristics of each group of tumors. *P* < 0.05. Scale bar = 100 μm.

To investigate the impact of varying modes of administration and dosage on animal models, we employed three distinct induction modalities and noninvasive monitoring using [18F]FASu and [18F]FDG (**Figure** 2A). The results showed that there was no significant difference in the incidence of hepatocellular carcinoma among the three induction modalities. However, there was a greater number of nodules and maximum nodule diameter in the 80 mg/kg i.g. group (**Figure** 2B-C and **Figure** S3). Pathological examination revealed moderate to high expression of AFP and Ki-67 in all three groups, indicating the formation of hepatocellular carcinoma (HCC). Anatomical drawings demonstrated a rougher surface of the liver with the presence of small irregular nodules. Pathological sections indicated a large number of hepatocytes with degeneration and atypical hepatocytes with extensive trabeculae in the center of the tumor with hemorrhagic and ischemic necrosis. Representative *ex vivo* photographs aligned with the previous quantification and characteristics of each group of tumors (**Figure** 2D).

### [18F]FASu monitored early oxidative stress damage during DEN induction

**Figure 3:**
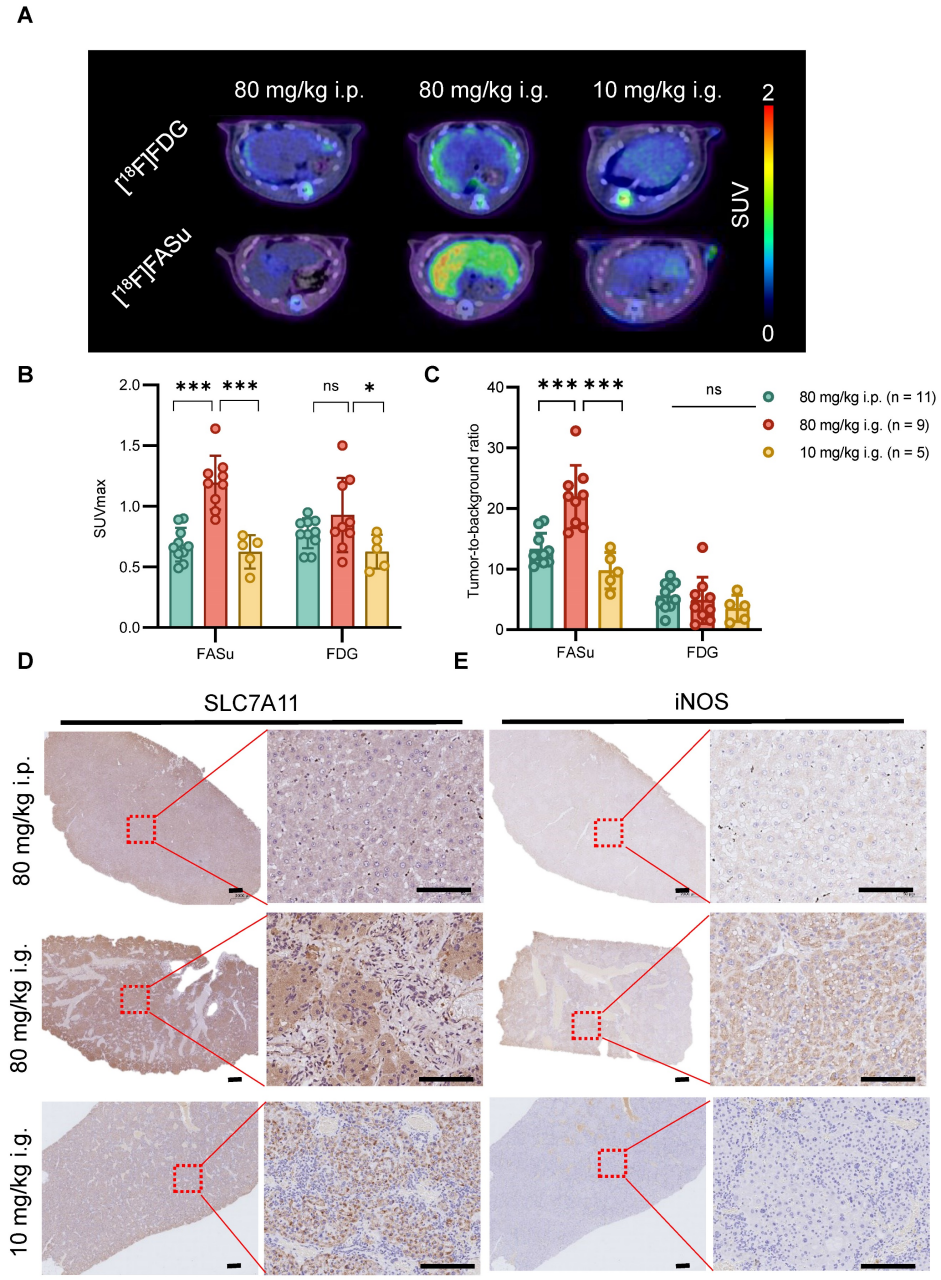
At Week 8, *in vivo* [18F]FASu can reveal the differential progression of HCC in three different delivery modes groups with DEN-induced rats.** (A)** Typical transverse images of [18F]FASu and [18F]FDG PET/CT in different groups at 1h after injection. Standardized uptake value max (SUVmax) **(B)** and TBR **(C)** of liver. We analyzed the SLC7A11 **(D)**, and iNOS **(E)** pathology results in each group around week 8. The liver of rats in the 80 mg/kg i.g. group showed significant inflammatory infiltration compared to the other two groups, while the corresponding SLC7A11 was highly expressed. ns, not statistically significant. ***P < 0.001. Scale bar = 1000 μm.

At Week 8, among the three groups of rats with different dosing regimens, PET imaging in the 80 mg/kg i.p. group showed a significantly high specific uptake of [18F]FASu (**Figure** 3A). The quantitative analysis showed that the [18F]FASu SUVmax of the 80 mg/kg i.g. group was 1.76 and 1.94 folds higher than that of the other two groups (80 mg/kg i.p.: 0.68 ± 0.13, 80 mg/kg i.g.: 1.20 ± 0.22, 10 mg/i.g.: 0.62 ± 0.13, *P* < 0.05). While no differential uptake of intrahepatic tumors was observed in the images using clinically used [18F]FDG, quantitative analyses revealed that [18F]FDG SUVmax values were statistically different only at 80 mg/kg i.g. and 10 mg/kg i.g. (80 mg/kg i.p.: 0.78 ± 0.12, 80 mg/kg i.g.: 0.93 ± 0.31, 10 mg/kg i.g.: 0.93 ± 0.13, *P* < 0.05) (**Figure** 3B). The tumor-to-background ratio indicated a higher specificity of [18F]FASu in the 80 mg/kg i.g. group, whereas [18F]FDG demonstrated no superior specific uptake in the three different groups of treated rats (**Figure** 3C).

To investigate the effects of different administration modes on the liver at an early stage, we performed pathological analysis on the livers of rats in each group at Week 8 and found that the expression of SLC7A11 and iNOS (inducible Nitric Oxide Synthase, an inflammatory marker) was significantly higher in the 80 mg/kg i.g. group, which indicated that they were in different degrees of hepatic injury (**Figure** 3D-3E). Similarly, the expression of 8-OHdG (an oxidative stress marker) was significantly higher in 80 mg/kg i.g. than in the other two groups, however the expression of γ-H2AX (a DNA damage marker) was higher in 80 mg/kg i.p. (**Figure** S5). In summary, these data suggest that the different delivery routes of DEN result in varying degrees of liver injury and oxidative stress which can be detected with [18F]FASu.

Finally, we also verified the specific uptake of reactive oxygen species (ROS) by [18F]FASu and [18F]FDG, and the uptake of [18F]FASu was significantly elevated in cells under oxidative stress conditions (**Figure** S4).

### The sensitivity and detection rate of [18F]FASu in HCC

**Figure 4:**
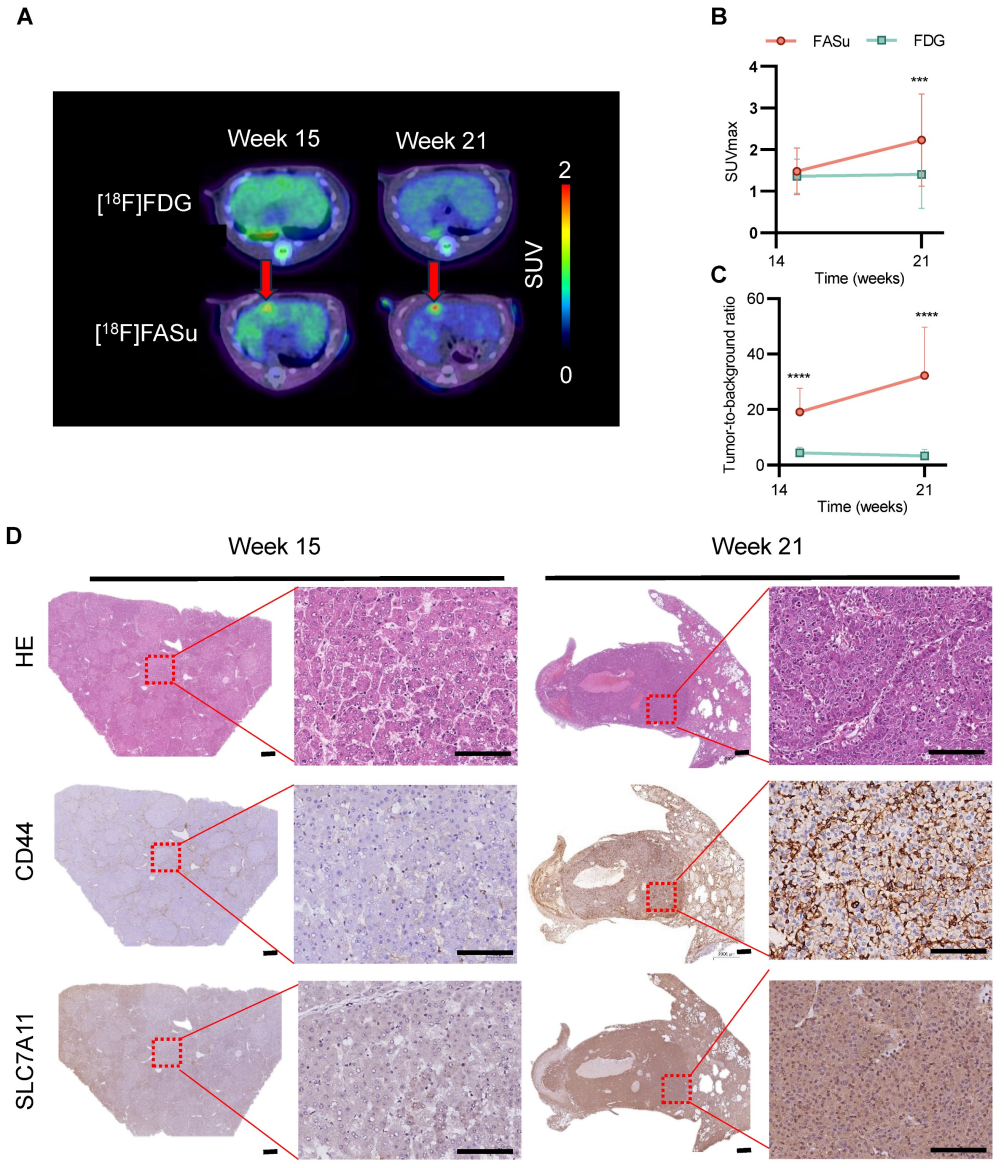
[18F]FASu enables early diagnosis of tumorigenesis. **(A)** PET/CT imaging of [18F]FASu and [18F]FDG in DEN-induced primary HCC rats at different time points. Rats were intravenously injected with 7.4 MBq of [18F]FASu and scanned at 1 h postinjection. Tumor lesions were pointed out by red arrows. Comparison of SUVmax **(B)** and TBR **(C)** of time-dependent [18F]FASu and FDG for all tumor lesions. **(D)** Pathologic results showed a time-dependent expression of CD44 and SLC7A11, both of which were significantly higher at Week 21 than at Week 15. **P < 0.001. ***P < 0.0001. Scale bar = 1000 μm.

The pathophysiology of rat liver underwent time-dependent changes with increasing DEN induction time and was monitored with [18F]FASu and [18F]FDG, respectively. We found that [18F]FASu was able to detect focal uptake at Week 15, and the SUVmax value of the tumor was further increased at Week 21, which could not be observed by [18F]FDG (**Figure** 4A). Analysis of SUVmax and tumor-to-muscle ratio (TBR) of [18F]FASu and [18F]FDG at the lesion site at three time points revealed that at Week 21, the former had significantly higher SUVmax values than the latter. Owing to the low liver background of [18F]FASu, the TBR of [18F]FASu was significantly higher than that of [18F]FDG at all three time points (**Figure** 4B-4C). Analysis of the pathological findings at the corresponding time points revealed moderate or low expression of CD44 and SLC7A11 at Week 15 and significantly enhanced expression of CD44 and SLC7A11 at Week 22 (**Figure** 4D).

**Figure 5:**
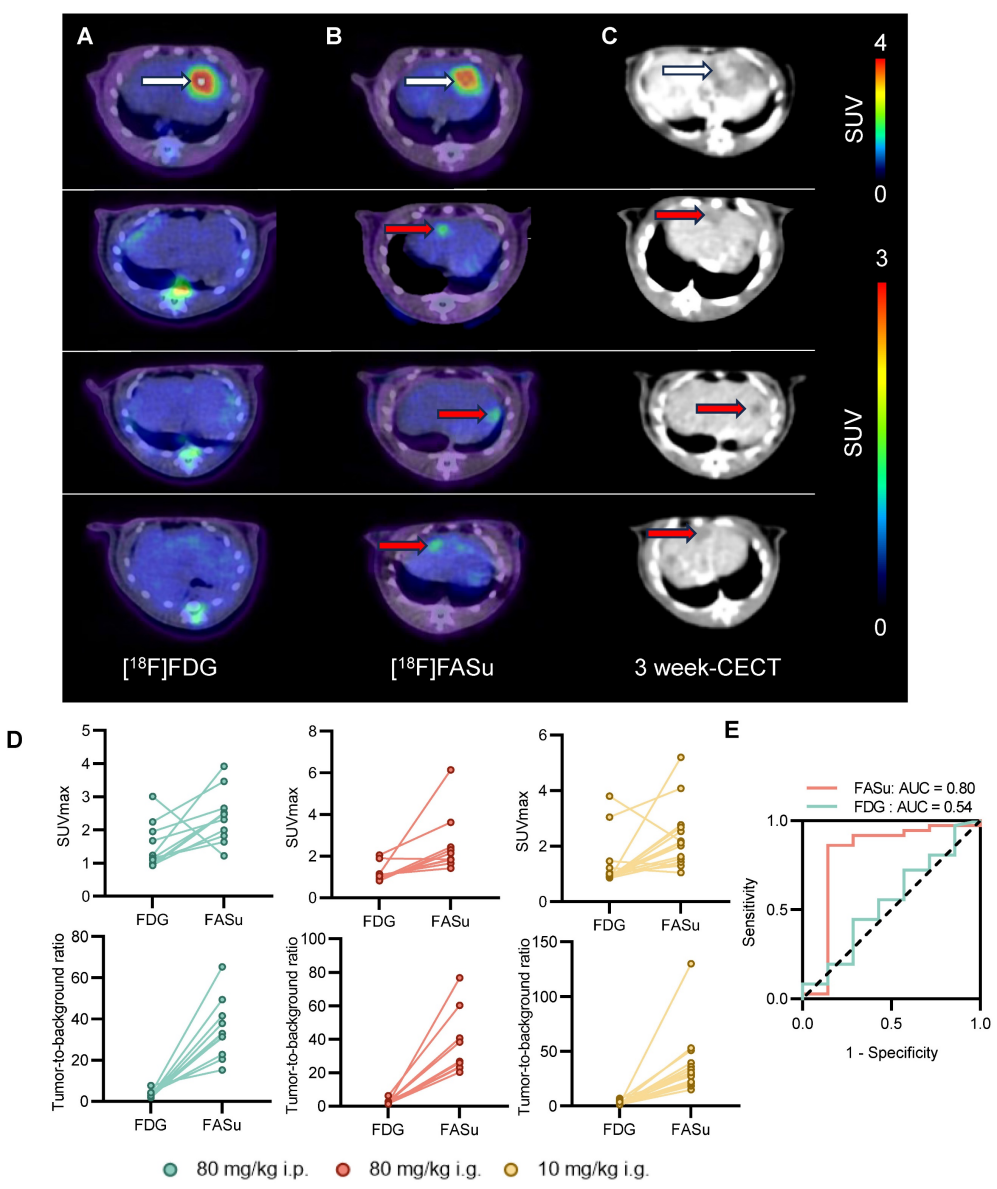
[18F]FASu has a higher sensitivity than [18F]FDG. At Week 21, [18F]FASu PET/CT **(A)**, [18F]FDG PET/CT **(B)**, and CECT **(C)**. [18F]FASu PET/CT consistently showed more lesions than [18F]FDG PET/CT. white arrowheads indicate lesions detected by [18F]FASu and [18F]FDG, and red arrowheads indicate lesions detected only by [18F]FASu (confirmed by their appearance at corresponding locations in the liver on CECT performed 3 weeks later). **(D)** For quantitative analysis of SUVmax and TBR for all tumors. **(E)** An ROC curve showed the better diagnostic performance of FASu over FDG.

At Week 21, PET images indicated that [18F]FASu detected more lesions compared to [18F]FDG, and because [18F]FASu is not dependent on glucose activity, there was less background signal in the brain, liver, oral and nasopharyngeal mucosa, or gastrointestinal tract compared to [18F]FDG. In detecting pathologically confirmed HCC lesions in 14 rats, the tumor detection rate with [18F]FASu by visual assessment was 94.4% (34/36), whereas that of [18F]FDG was 25% (9/36) (**Figure** 5A-5B, **Table** 1). Corresponding CECT images further confirmed the presence of the tumor (**Figure** 5C). In order to further quantify the signal intensity of the two probes and to facilitate the comparison of their uptake differences, the liver lesion uptake SUVmax of [18F]FASu and [18F]FDG was analyzed, and in agreement with the visualization results, the SUVmax value and TBR value of [18F]FASu were significantly higher than that of [18F]FDG in the lesions (**Figure** 5D). SUVmax of [18F]FASu provided an AUC of 0.80 (95%CI 0.55-1.00, *P* < 0.05) in the ROC analysis, with a sensitivity of 86.11% (95%CI 71.34%-93.92%) and specificity of 85.71% (95%CI 48.69%-99.27%) at the optimal cut-off point of 1.570; SUVmax of [18F]FDG provided an AUC of 0.54 (95%CI 0.29-0.78, *P* > 0.05) in the ROC analysis, with a sensitivity of 44.44% (95%CI 29.54%-60.42%) and specificity of 71.43% (95%CI 35.89%-94.92%) at the optimal cut-off point of 1.130 (**Figure** 5E). Thus, the ROC curve showed the better diagnostic performance of FASu over FDG.

### Correlation between [18F]FASu uptake and immunohistochemical staining of SLC7A11 and CD44

**Figure 6:**
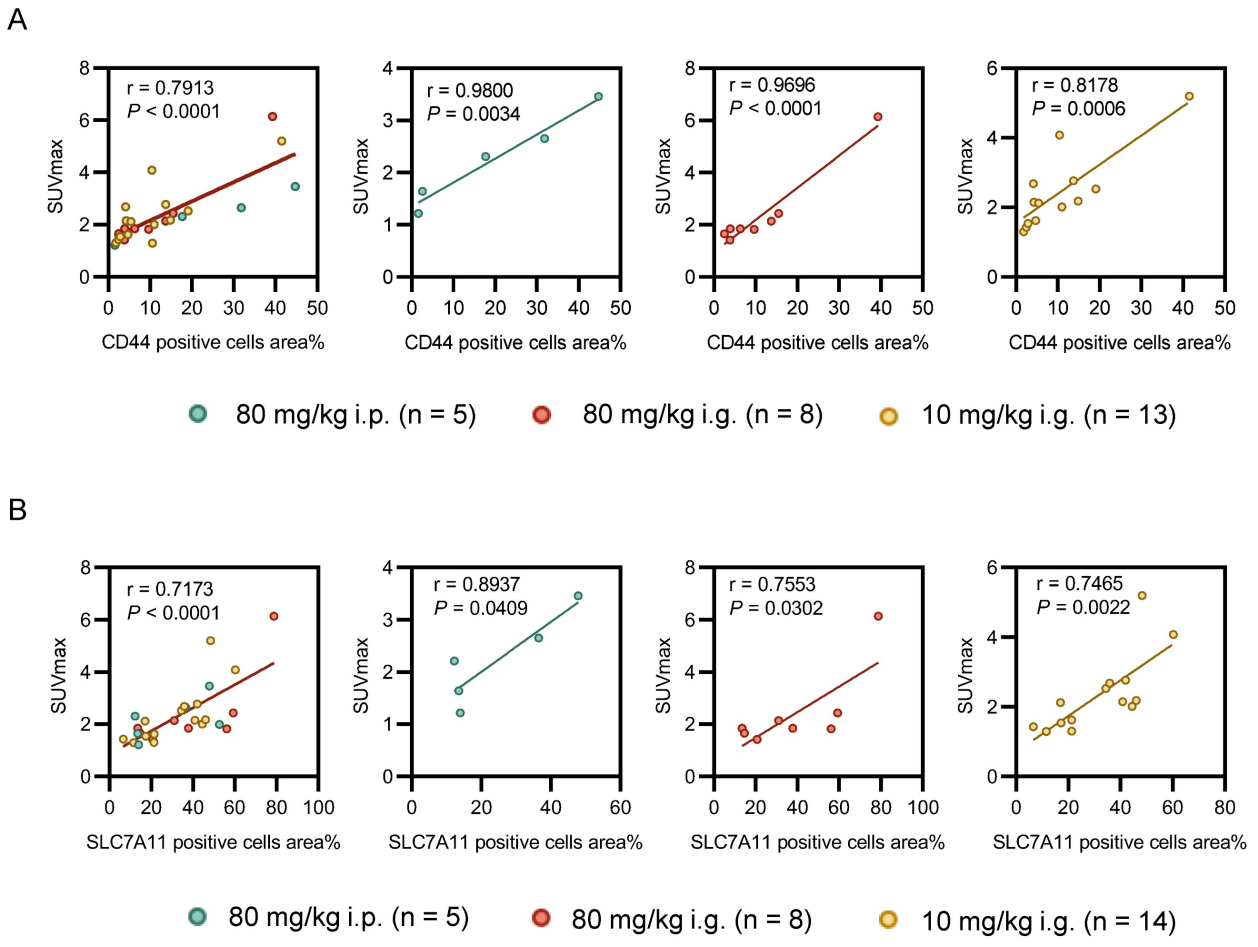
Correlation between pathology and oxidative stress levels reflected by [18F]FASu SUVmax at different doses. **(A)** There was a significant correlation between the percentage area of CD44-positive cells and [18F]FASu SUVmax across all groups (80 mg/kg i.p.: r = 0.9800, *P* = 0.0034, n = 5; 80 mg/kg i.g.: r = 0.9696, *P* < 0.0001, n = 8; and 10 mg/kg i.g.: r = 0.8178, *P* = 0.0006, n = 13). **(B)** Correlation between the percentage of SLC7A11-positive cells and the uptake of [18F]FASu (80 mg/kg i.p.: r = 0.8937, *P* = 0.0409, n = 5; 80 mg/kg i.g.: r = 0.7553, *P* = 0.0302, n = 8; and 10 mg/kg i.g.: r = 0.7465, *P* = 0.0022, n = 14).

Statistical analyses revealed a significant correlation of [18F]FASu SUVmax 60 minutes post injection with the percentage of CD44 positive cells area (**Figure** 6A, all tumors: r = 0.7913, *P* < 0.0001, n = 26; 80 mg/kg i.p.: r = 0.9800, *P* = 0.0034, n = 5; group 80 mg/kg i.g.: r = 0.9696, *P* < 0.0001, n = 8; and group 10 mg/kg i.g.: r = 0.8178, *P* = 0.0006, n = 13) and with percentage of SLC7A11 positive cells area (**Figure** 6B, all tumors: r = 0.7173 , *P* < 0.0001, n = 27; 80 mg/kg i.p.: r = 0.8937, *P* = 0.0409, n = 5; group 80 mg/kg i.g.: r = 0.7553, *P* = 0.0302, n = 8; and group 10 mg/kg i.g.: r = 0.7465, *P* = 0.0022, n = 14). No significant relationship was observed between [18F]FDG and immunohistochemical staining scores of SLC7A11 and CD44 (**Figure** S6, *P* > 0.05).

## Discussion

Current recommendations for HCC screening, as endorsed by professional society guidelines, include semi-annual abdominal ultrasound with or without serum alpha-fetoprotein (AFP) for patients with cirrhosis and subgroups with chronic hepatitis B virus infection 28. However, hepatic ultrasonography for early-stage HCC identification has a pooled sensitivity of 45%, increasing to 63% when alpha-fetoprotein (AFP) is included. Although computed tomography (CT) and magnetic resonance imaging (MRI) are more sensitive, they are associated with false positives, limiting their use as primary surveillance strategies 29. Non-invasive monitoring of glycogen metabolism using MRI has also been studied in recent years 30. While MR/CT imaging can provide structural information with high sensitivity, they have the most value after the tumor has already grown to a certain size. Thus, early detection of HCC remains an unmet need in clinical practice for diagnosis, staging, and treatment planning. The etiology and the chronic process of HCC formation highlight the potential for establishing specific features of oxidative-stress metabolism during HCC formation for early diagnosis and underscore the importance of animal models for longitudinal characterization during HCC formation. The model of DEN-induced HCC in rats largely recapitulates the pathogenesis of HCC seen in humans 31, including the differential activation of proteins involved in oxidative stress and cell damage during the progression of carcinogenesis 32. Here, we evaluated whether the process of HCC formation in DEN-induced rat models could be detected by oxidative stress-related [18F]FASu PET imaging and compared the PET imaging feature of [18F]FASu PET with [18F]FDG PET. Bioinformatics analysis confirmed that SLC7A11, the light chain in the system x_C_-, was closely associated with HCC and was significantly upregulated in HCC patients and DEN-induced rat models by Kaplan-Meier analysis. To identify the effects of different administration ways and dosages of DEN on HCC generation, longitudinal noninvasive [18F]FASu, and [18F]FDG PET were used in three induction modalities, 80 mg/kg i.p., 80 mg/kg i.g., 10 mg/i.g. Higher uptake and higher sensitivity of [18F]FASu were confirmed at early status (at Week 8 post administration of DEN) and final status (at Week 21 post administration of DEN), respectively, as compared to [18F]FDG PET. Statistical analyses revealed a significant correlation of [18F]FASu SUVmax with SLC7A11 transporter (r = 0.71, P < 0.0001). Overall, utilizing a PET tracer targeting SLC7A11 with [18F]FASu showed higher sensitivity in diagnosing HCC and enabled the early-stage characterization of pathological changes in DEN-induced HCC rats.

### Oxidative stress altered cellular metabolism allowing for the early detection of HCC formation

Altered cellular metabolism due to changes in cell niches during homeostasis can impair tissue and organ function, ultimately contributing to disease. In particular, oxidative stress towards hepatic cells directly leads to liver tissue damage and promotes the initiation and progression of HCC 33. In the realm of oxidative stress-induced diseases, accumulated evidence has underscored the increasingly significant role of the cystine/glutamate transporter system x_C_- in cell growth, proliferation, metastasis, and multidrug resistance across various cancer types 34. system x_C_- consists of a light-chain subunit (xCT, SLC7A11) and a heavy-chain subunit (CD98hc, SLC3A2), forming a cystine/glutamate exchange transporter 35. SLC7A11 is crucial for maintaining intracellular cysteine balance and facilitating the biosynthesis of GSH biosynthesis, the main intracellular antioxidant component 36. During oxidative stress, expression of the antioxidant response-regulated gene SLC7A11 is upregulated through the KEAP1-Nrf2 pathway 27. Further, SLC7A11 is frequently overexpressed in various cancers, including HCC, and its upregulation can facilitate tumor growth 33.

Although numerous HCC rat models have been described in the literature 37-39, different administration routes and dosages of chemical carcinogen exposure utilized to generate the tumors make it difficult to pinpoint the factors influencing tumor formation. Our findings suggest that a relatively high DEN dose (80 mg/kg) leads to an increase in the number of nodules compared to the 10 mg/kg dose group (**Figure** S3). In particular, 80 mg/kg i.g. resulted in the formation of larger nodules (>15 mm) (**Figure** 2). Moreover, at the early time point post-DEN administration (Week 8), the livers of rats in the 80 mg/kg i.g. group exhibited significant inflammatory infiltration compared to the other two groups, with a corresponding high expression of SLC7A11 (**Figure** 4). In the gastrointestinal tract, many enzymes allow the absorption and transport of DEN into the liver, which may lead to a higher and quicker accumulation of DEN in rat liver. That may explain the more aggressive oxidative stress for rat liver in 80 mg/kg i.g. group. Thus, the oxidative stress induced by DEN led to the upregulation of the cellular antioxidant pathway, which altered cellular metabolism. It may allow for the early detection of the dysplastic niche associated with HCC formation.

### PET imaging with SLC7A11 targeting [18F]FASu tracer demonstrated higher sensitivity and tumor detection rate in rat HCC

18F-5-fluoro-aminosuberic acid ([18F]FASu) is a PET radiotracer for monitoring system x_C_- activity from oxidative stress 40. This study found that [18F]FASu has higher sensitivity than [18F]FDG in detecting DEN-induced HCC rat tumors. Interestingly, early on in the 80 mg/kg i.g. group (Week 8), there was a diffuse high uptake of [18F]FASu in the liver compared to the other two groups. In contrast, this difference was barely observable for [18F]FDG. SLC7A11 is less expressed in normal liver tissue while upregulated in repopulating hepatocytes after liver injury (e.g., drug-induced injury and toxin-induced injury), possibly protecting them from oxidative stress and promoting regeneration 41, 42. Further, SLC7A11 plays a vital role in the pathogenesis of HCC, including liver damage, chronic inflammation, hepatocyte proliferation, liver fibrosis and cirrhosis, disorganized vasculature, and modulations of the liver's immune microenvironment 31. Our results suggest that [18F]FASu has excellent potential for diagnosing chronic hepatitis or liver fibrosis prior to the HCC formation.

### Limitations

Our findings provide insight into early detection of HCC through oxidative stress-related PET imaging in rat HCC models. The most important limitation is the lack of patient data to correlate with the results of the animal models. Despite this, as DEN-induced HCC involving oxidative stress is similar to the clinical HCC caused by chronic hepatitis, oxidative stress-related PET imaging may still be valuable in early HCC detection. Another limitation is that only three groups with different administration methods and dosages of DEN were used to differentiate oxidative stress levels. Moreover, although the longitudinal evaluation of dynamic features during the development of DEN-induced rat HCC by [18F]FASu PET/CT imaging, the lack of parallel monitoring of tumor histology after DEN administration may obscure other pathological features. Furthermore, some of the small nodules in the *ex vivo* examination of rat HCC were missed on the PET/CT. Lastly, as our work focused on imaging system x_C_- activity after the induction of oxidative stress to detect HCC, a more specific tracer, FASu, was used in preference to FSPG 43, 44. In the work reported by Webster *et al.*, sulfasalazine, a system x_C_- specific inhibitor, was superior to ʟ-glutamate in preventing [18F]FASu accumulation 26. In contrast, the other glutamate receptors and transporter mechanisms involving the cellular uptake of [18F]FSPG may complicate *in vivo* pharmacokinetics 43. Therefore, in our work, [18F]FASu was used for imaging system x_C_-. However, it may be interesting to compare the images of FASu to FSPG to detect HCC in the future.

## Conclusion

In our study, compared to the conventional [18F]FDG tracer, [18F]FASu PET imaging demonstrated higher sensitivity in diagnosing HCC and enabled early-stage characterization of oxidative stress in DEN-induced rat HCC. The findings regarding the oxidative stress characterization of HCC formation in DEN-induced rat models using [18F]FASu PET imaging provide new horizontals for HCC diagnosis.

## Supplementary Material

Supplementary methods and figures.

## Figures and Tables

**Table 1:** Comparison of [18F]FDG and [18F]FASu uptake at 60 min after injection in rats with HCC

Parameter	[18F]FDG	[18F]FASu	*P*
Qualitative sensitivity	25% (9/36)	94.4% (34/36)	< 0.001
Qualitative analysis			
SUVmax	1.33 ± 0.71	2.38 ± 1.10	< 0.001
SUVmean of muscle	0.54 ± 0.21	0.07 ± 0.01	< 0.001
TBR	2.80 ± 1.66	35.76 ± 21.62	< 0.001

## Abbreviations

HCC: hepatocellular carcinoma
PET: positron emission tomography
CT: computed tomography
DEN: diethylnitrosamine
[18F]FASu: 18F-5-fluoroaminosuberic acid
[18F]FDG: 18F-fluorodeoxyglucose
FAP: fibroblast activating protein
PSMA: prostate-specific membrane antigen
SLC7A11: solute carrier family 7a member 11
GPX4: glutathione peroxidase 4
GSH: glutathione
RCY: radiochemical yield
H&E: hematoxylin & eosin
AFP: alpha-fetoprotein
BSA: bovine serum albumin
CECT: contrast-enhanced CT
SD: standard deviation
TBR: tumor-to-muscle ratio
DFI: disease-free interval
MRI: magnetic resonance imaging
OS: overall survival
LH: liver hepatitis
LC: liver cirrhosis
NC: normal control
ROS: reactive oxygen species. ROC: Receiver Operating Characteristic
AUC: Area Under the Curve
