# Supplementary material for: 18F-5-fluoro-aminosuberic acid PET/CT imaging of oxidative-stress features during the formation of DEN-induced rat hepatocellular carcinoma
Source: Theranostics. 2025 Jan 1;15(1):141–54. doi: 10.7150/thno.100467 (PMC11667236; doi:10.7150/thno.100467)
Supplement: Supplementary file 1 — Supplementary methods and figures. [file thnov15p0141s1.pdf]

Supplementary Information

^{18}F-5-fluoro-aminosuberic acid PET/CT imaging of oxidative-stress features during the formation of DEN-induced rat hepatocellular carcinoma

Feng Xiong[1,2], Yilin Yang[1,2], Zhengru Han[3], Buchuan Zhang[1,2], Kijung Kwak[4], Pei Wang[1,2], Qiaorong Chen[1,2], Ziqiang Wang[1], Jingfei Yang[1], Xiaoyun Deng[1], Sijuan Zou[1], Zhuoli Zhang[5], Pengtao You*[3], Bo Yu*[1,2], Xiaohua Zhu*[1]

1 Department of Nuclear Medicine, Tongji Hospital, Tongji Medical College, Huazhong University of Science and Technology, Wuhan, China.

2 Department of Nuclear Medicine, Tongji Hospital, Tongji Medical College and State Key Laboratory for Diagnosis and Treatment of Severe Zoonotic Infectious Diseases, Huazhong University of Science and Technology, Wuhan, China.

3 Hubei University of Chinese Medicine, Wuhan 430030, Hubei Province, China.

4 University of Maryland School of Medicine, 655 W Baltimore St S, Baltimore, MD 21201

5 Department of Radiological Sciences, University of California Irvine, CA, USA.

Corresponding authors:

Prof. Bo Yu, PhD

yuboxf@hotmail.com

Prof. Xiaohua Zhu, MD PhD

evazhu@vip.sina.com

Prof. Pengtao You, PhD

tptyou@hbucm.com

Characterization of [^{18}F]FASu

HPLC confirmed the identities of [^{18}F]FASu. Quality control of the radiosynthesis was performed by ultraviolet and radio-HPLC. The chromatograms are shown in Supplemental Figure 1. [^{18}F]FASu was characterized on reverse phase HPLC using Waters C18 column (5 mm, 250 mm × 4.6 mm) at a constant flow rate of 1 mL/min. The product was eluted using a gradient mobile phase from 0% solvent B at 0 min to 15% solvent B at 20 min: solvent A = 0.1% trifluoroacetic acid (TFA) in H2O; solvent B = acetonitrile.

Determination of chirality

For the products synthesized, HPLC characterization using a d-penicillamin column with CuSO4 (aq.) eluent, a technique known to separate L- and D-isomers of amino acids with the L-isomer eluting first [1], showed two sets of peaks for [^{18}F]FASu (Figure S2). Further, the phase-transfer catalysts (PTC) were altered to produce two other compounds with different chiral properties (L-[^{18}F]FASu: D-[^{18}F]FASu = 100:0, L-[^{18}F]FASu: D-[^{18}F]FASu = 64 :36) [2], and the high uptake of the 2S-configuration (also named L-[^{18}F]FASu) was again verified. Specifically, the PTC was altered to 18-crown-6/$KHCO_3$ and the solvent used MeCN, and the reaction was carried out at 70°C for 15 min to obtain a product of L-[^{18}F]FASu: D-[^{18}F]FASu = 100:0. Similarly, the PTC was changed to K2.2.2/$KHCO_3$ and the solvent was used DMSO, and the reaction was carried out at 95°C for 20 min to obtain a product of L-[^{18}F]FASu: D-[^{18}F]FASu = 64:36.

Cell culture and uptake experiments

The hepa1-6 cell line was cultured in complete Dulbecco modified Eagle medium (with glucose + 10% fetal bovine serum + 1% penicillin/streptomycin, DMEM). Cells were maintained in 75 cm^2 flasks in a humidified incubator at 37°C with 5% CO2 and routinely subcultured at approximately 95% confluency. For uptake studies, cells were seeded into 6-well plates (4 × 10^5 cells/well) such that confluency was achieved the

next day. Each plate was washed 3 times with cold PBS, and then 0.148 MBq of different chiral [^{18}F]FASu were added. After incubation, supernatants were removed, cells were washed three times with cold PBS, 1000 mL of 1 M NaOH were added to the cells, the NaOH lysate was collected 1 min later. Counts per minute (CPM) was determined by using the gamma counter.

In vitro stability

For the vitro stability study, 0.70 MBq of [^{18}F]FASu was incubated at 37°C with 300 μL of phosphate-buffered saline (PBS) or fetal bovine serum (FBS) for 30, 60, 120 and 240 min. After each incubation, the sample was centrifuged through a 0.22-mm membrane and analyzed by radio-HPLC. The tracer was stable in PBS and FBS.

Immunohistochemical assay

The fresh tissue was fixed with a solution for more than 24 hours. The tissue was removed from the fixed solution after fixation. Then, the tissue was smoothed by using a scalpel in the fume hood and put into the embedding frame. The tissue was put into tap water and washed for 20 minutes. Tissue processing: Embedding frames containing tissue were put into the cassette and dehydrated with gradient ethanol in turn. The wax-soaked tissue was embedded by using tissue embedder. Firstly, melted paraffin was put in the bottom of the embedded mold. Then, tissue was taken out of the embedding frame when the paraffin solidified slightly and put into an embedded mold according to the requirements of the different sections. Tweezers were used to gently press the tissue to make the tissue completely flat at the bottom of the mold. Finally, the mold with tissue in it was covered by an embedding frame with a corresponding identifier and moved to the -20°C freezing table for cooling. The paraffin block was taken out from the mold after paraffin solidified. The trimmed paraffin block was put on the microtome to be sliced with 4μm thickness. Sections were floated on warm water (42°C) to be flatten and were picked up vertically by using a glass slide. After the tissue was dried, sections were put into an incubator at 60°C for 30 minutes to 1 hour. Sections were taken out of the incubator and stored at room temperature.

ROS measurement

Diethyl maleate (DEM) is a maleate ester resulting from the formal condensation of both carboxy groups of maleic acid with ethanol. DEM depletes glutathione (GSH) in exposed cells [3]. H2DCFDA (MCE, HY-D0940) is a cell-permeable probe used to detect intracellular reactive oxygen species (ROS) (Ex/Em = 488/525 nm) [4]. We first verified the ability of DEM to induce ROS by immunofluorescence experiments, treatment of hepa1-6 cells with 0.1 mM DEM for 1h and washed three time, then incubated with DMEM medium containing H2DCFDA (5 μM; 30 min) at 37 °C. The cells then are washed three times with warm DMEM medium. Subsequent observation under a fluorescence microscope (Olympus CKX53).

In diethyl maleate (DEM)-induced OS experiments, 0.1 mM of DEM was added 16 h and 1 h before harvest. The uptake study was conducted using the method described above.

Table S1. Comparison of the number and maximum diameter of nodules formed by different induction methods

	Total number of nodules	Maximum diameter	Number of nodules (＜ 0.5 mm)
80 mg/kg i.p. (n = 6)	29	4	12
80 mg/kg i.g. (n = 3)	49	21	15
10 mg/kg i.g. (n = 5)	14	5	5

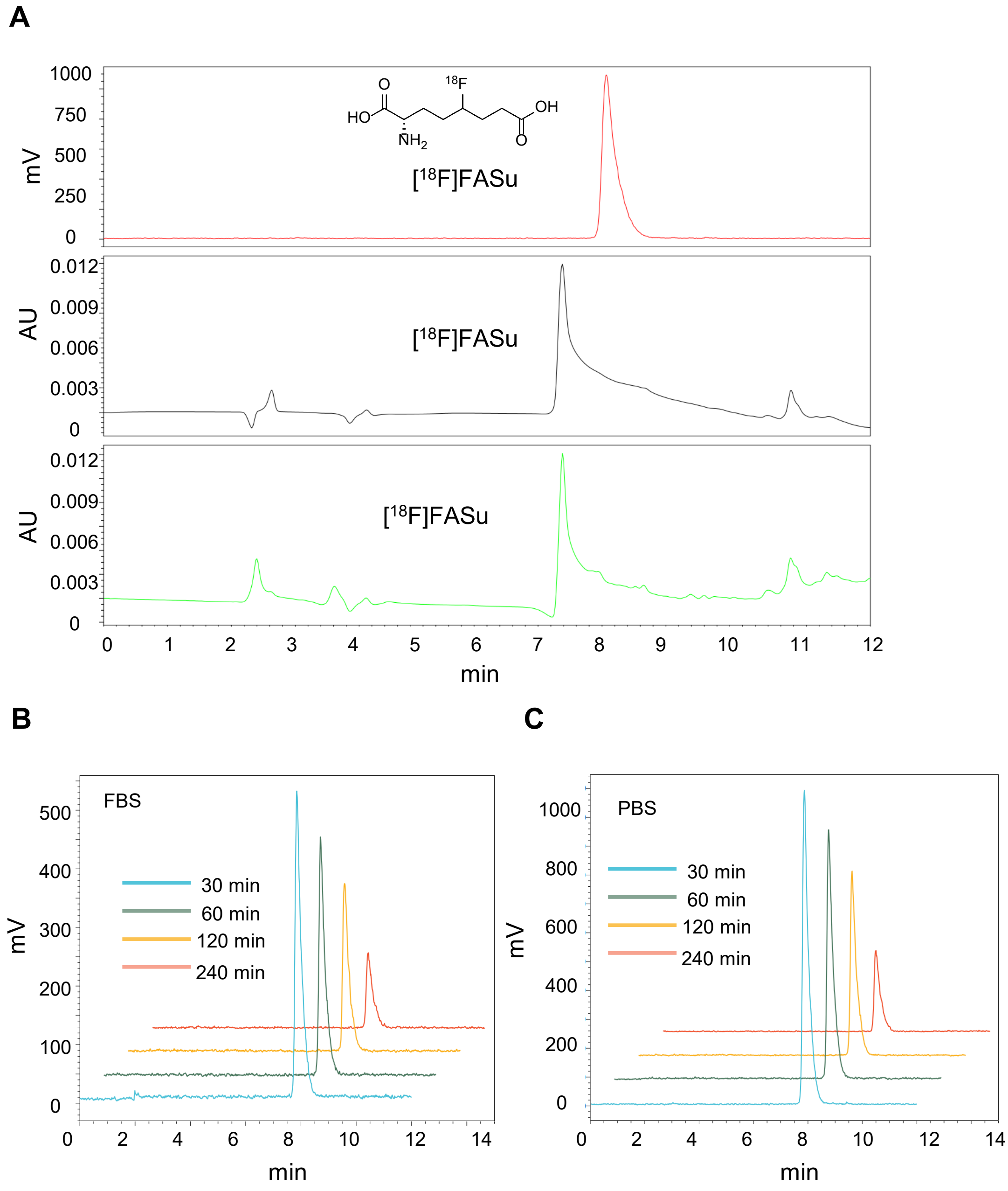

Figure S1. Characterization and in vitro stability of [^{18}F]FASu. (A) [^{18}F]FASu was characterized on reverse phase HPLC using Waters C18 column . γ tracer (top), UV 280 nm (middle), UV 256 nm (bottom). In vitro stability of [^{18}F]FASu in FBS (B) and PBS (C), respectively.

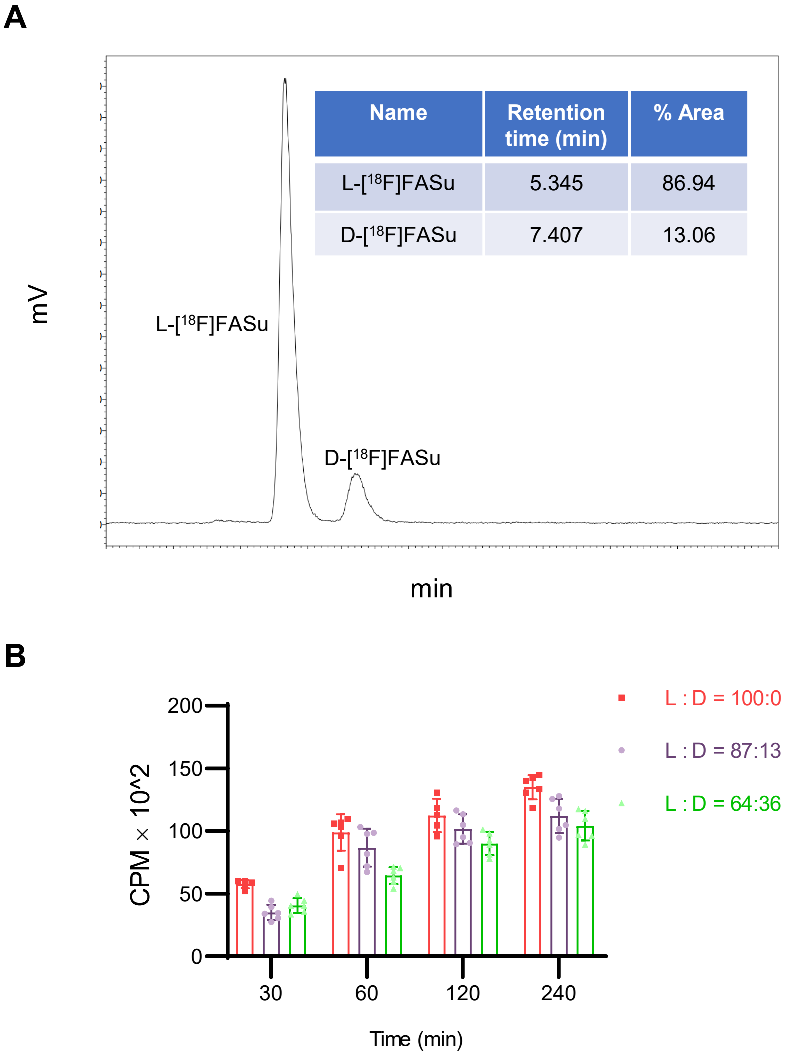

Figure S2. Chiral HPLC analysis and cellular uptake of different chiral products. (A) [18F]FASu was characterized on reverse phase HPLC using Phenomenex d-penicillamin column (5 mm, 250 mm × 4.6 mm) with CuSO4 (aq.,1 mM)/EtOH = 85:15, isocratic at a constant flow rate of 1 mL/min. (B) Three chiral [^{18}F]FASu were incubated with hepa1-6 cells for 30, 60, 120, and 240 min, respectively.

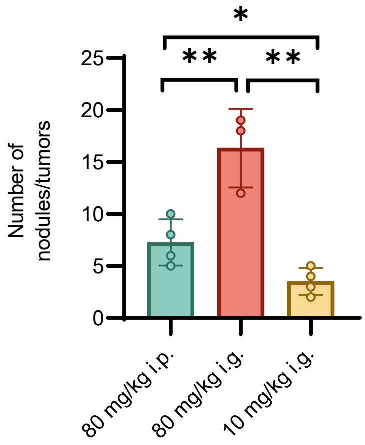

Figure S3. Liver visualization to assess the number of tumors on the surface of the liver. *$P < 0.05$, **$P < 0.01$.

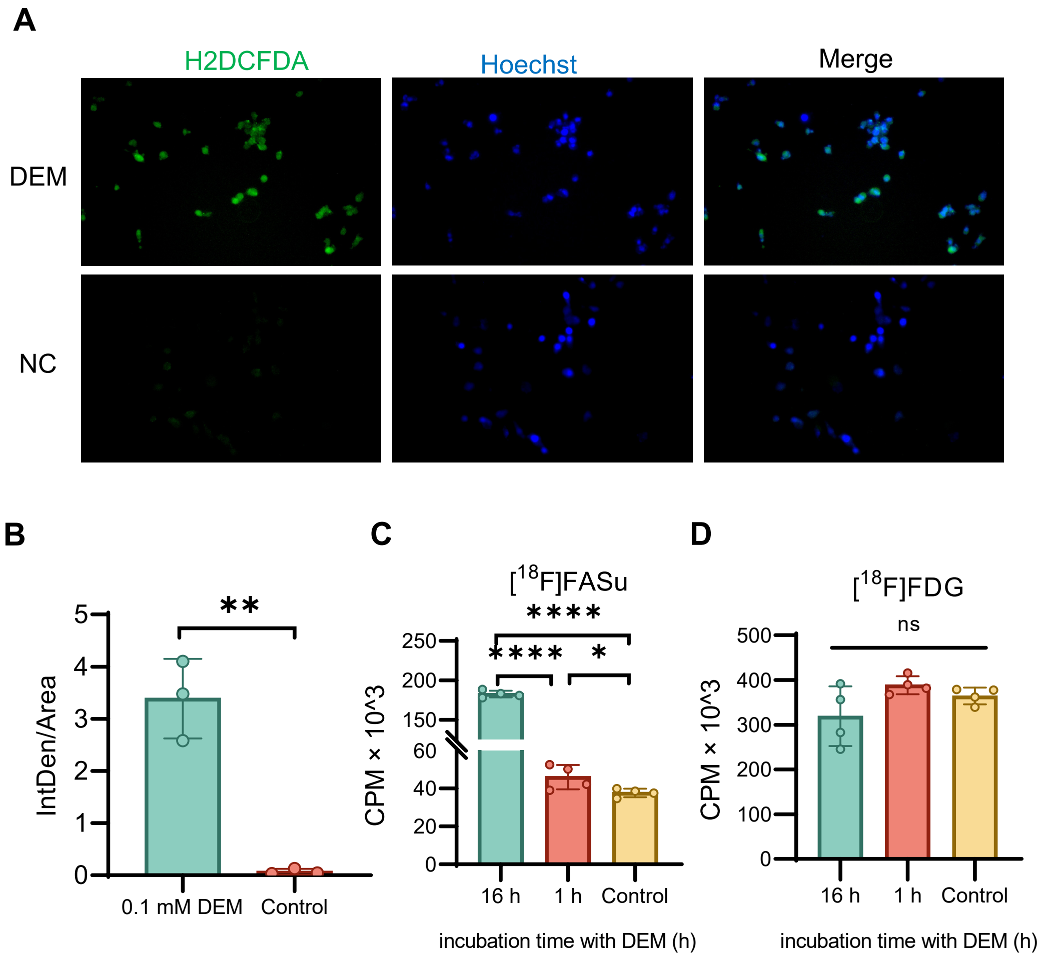

Figure S4. [^{18}F]FDG and [^{18}F]FASu response to ROS. **(A)** In vitro experiments are conducted to measure the cellular ROS levels in hepa1-6 cells from 0.1 mM DEM treatments using fluorescent probe H2DCFDA (5 µM; 30 min) (NC = Normal control). **(B)** Semi-quantitative results of fluorescence. Cellular uptake of [^{18}F]FASu **(C)** and [^{18}F]FDG **(D)** for hepa1-6 cells treated for different times with 0.1 mM DEM. ns, not statistically significant. *$P < 0.05$, **$P < 0.01$, ****$P < 0.0001$.

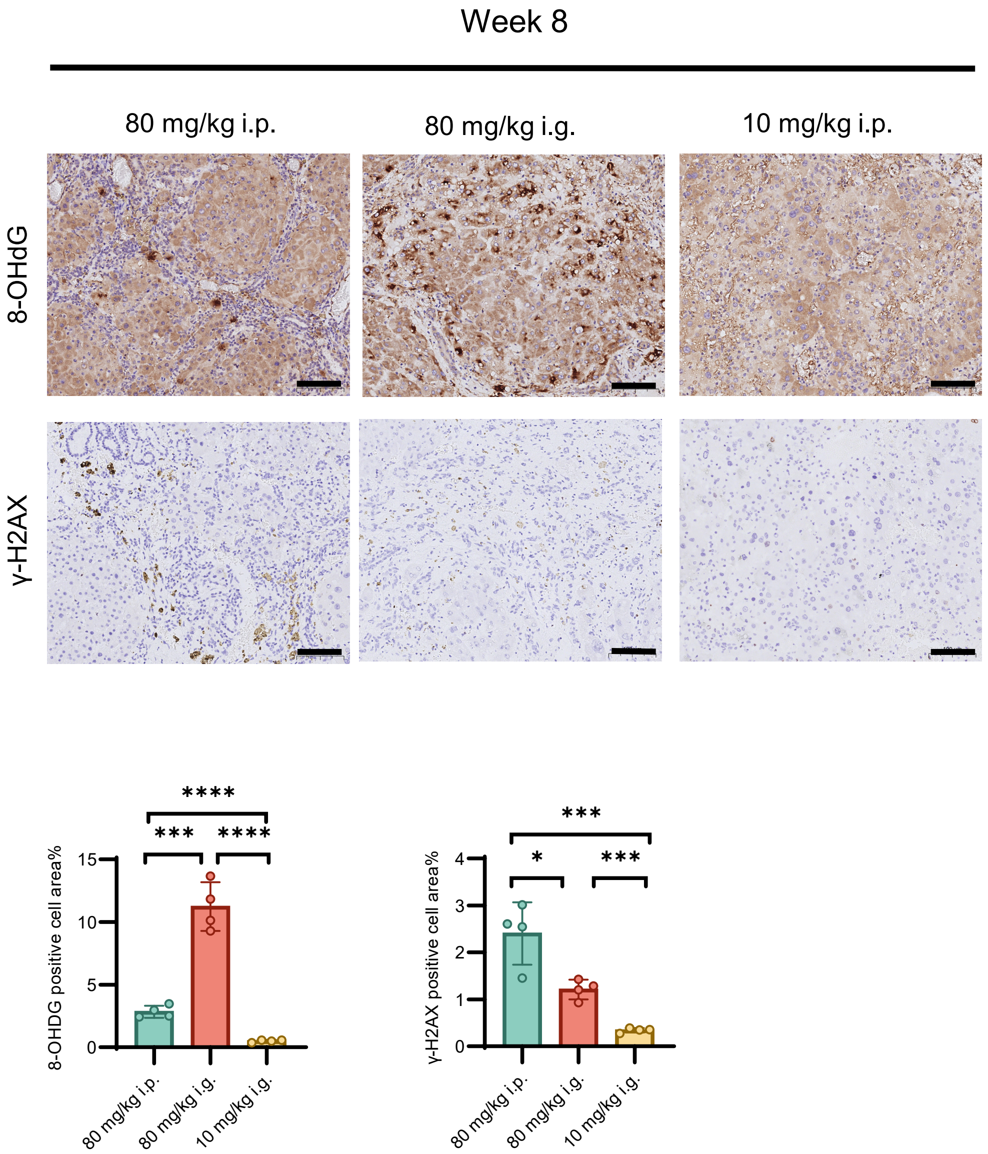

\

Figure S5. Levels of oxidative stress in rats induced by DEN. ns, not statistically significant. *$P < 0.05$, ***$P < 0.001$, ****$P < 0.0001$. Scale bar = 100 μm.

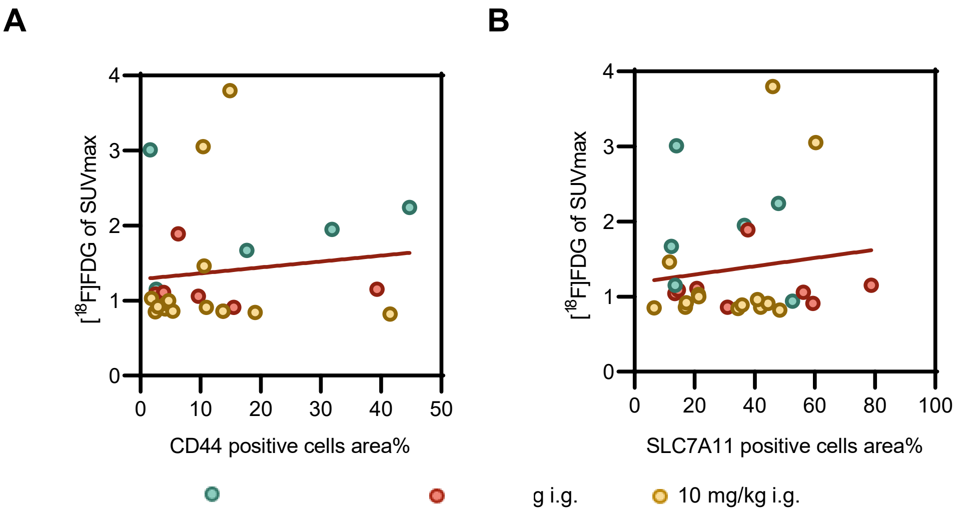

Figure S6. Correlation between SUVmax of [^{18}F]FDG and expression of CD44 and SLC7A11.

1. Qu W, Zha Z, Ploessl K, Lieberman BP, Zhu L, Wise DR, et al. Synthesis of optically pure 4-fluoro-glutamines as potential metabolic imaging agents for tumors. J Am Chem Soc. 2011; 133: 1122-33.
2. Yang H, Tam B, Colovic M, Southcott L, Merkens H, Benard F, et al. Addressing Chirality in the Structure and Synthesis of [(18) F]5-Fluoroaminosuberic Acid ([(18) F]FASu). Chemistry. 2017; 23: 11100-7.
3. Gille A, Turkistani A, Tsitsipatis D, Hou X, Tauber S, Hamann I, et al. Nuclear trapping of inactive FOXO1 by the Nrf2 activator diethyl maleate. Redox Biol. 2019; 20: 19-27.
4. Lyublinskaya OG, Ivanova JS, Pugovkina NA, Kozhukharova IV, Kovaleva ZV, Shatrova AN, et al. Redox environment in stem and differentiated cells: A quantitative approach. Redox Biol. 2017; 12: 758-69.
